# Development and validation of a predictive model for incident type 2 diabetes in middle-aged Mexican adults: the metabolic syndrome cohort

**DOI:** 10.1186/s12902-019-0361-8

**Published:** 2019-04-28

**Authors:** Olimpia Arellano-Campos, Donaji V. Gómez-Velasco, Omar Yaxmehen Bello-Chavolla, Ivette Cruz-Bautista, Marco A. Melgarejo-Hernandez, Liliana Muñoz-Hernandez, Luz E. Guillén, Jose de Jesus Garduño-Garcia, Ulices Alvirde, Yukiko Ono-Yoshikawa, Ricardo Choza-Romero, Leobardo Sauque-Reyna, Maria Eugenia Garay-Sevilla, Juan Manuel Malacara-Hernandez, Maria Teresa Tusie-Luna, Luis Miguel Gutierrez-Robledo, Francisco J. Gómez-Pérez, Rosalba Rojas, Carlos A. Aguilar-Salinas

**Affiliations:** 1 Unidad de Investigación de Enfermedades Metabólicas, Instituto Nacional de Ciencias Médicas y Nutrición Salvador Zubirán, Vasco de Quiroga 15, 14000 Mexico City, Mexico; 2 Departamento de Endocrinología, Metabolismo del Instituto Nacional de Ciencias Médicas y Nutrición Salvador Zubirán, Mexico City, Mexico; 3 MD/PhD (PECEM) Program, Facultad de Medicina, Universidad Nacional Autónoma de Mexico, Mexico City, Mexico; 4 Hospital General Regional No. 251, Instituto Mexicano del Seguro Social, Mexico City, Mexico; 5 Centro Médico Ono Aguascalientes, Mexico City, Mexico; 6 Unidad Metabólica y Cardiovascular, Cuernavaca, Morelos Mexico; 7 Instituto de Investigaciones Médicas, Universidad de Guanajuato, Leon, Guanajuato Mexico; 8 Unidad de Biología Molecular y Medicina Genómica, Instituto de Investigaciones Biomédicas, Mexico City, Mexico; 9 Instituto Nacional de Geriatria, Mexico City, Mexico; 10 Instituto Nacional de Salud Pública, Cuernavaca, Morelos Mexico

**Keywords:** México, Incidence, Obesity, Latinos, Urbanization, Diabetes prediction

## Abstract

**Background:**

Type 2 diabetes mellitus (T2D) is a leading cause of morbidity and mortality in Mexico. Here, we aimed to report incidence rates (IR) of type 2 diabetes in middle-aged apparently-healthy Mexican adults, identify risk factors associated to ID and develop a predictive model for ID in a high-risk population.

**Methods:**

Prospective 3-year observational cohort, comprised of apparently-healthy adults from urban settings of central Mexico in whom demographic, anthropometric and biochemical data was collected. We evaluated risk factors for ID using Cox proportional hazard regression and developed predictive models for ID.

**Results:**

We included 7636 participants of whom 6144 completed follow-up. We observed 331 ID cases (IR: 21.9 per 1000 person-years, 95%CI 21.37–22.47). Risk factors for ID included family history of diabetes, age, abdominal obesity, waist-height ratio, impaired fasting glucose (IFG), HOMA2-IR and metabolic syndrome. Early-onset ID was also high (IR 14.77 per 1000 person-years, 95%CI 14.21–15.35), and risk factors included HOMA-IR and IFG. Our ID predictive model included age, hypertriglyceridemia, IFG, hypertension and abdominal obesity as predictors (D_xy_ = 0.487, *c-statistic* = 0.741) and had higher predictive accuracy compared to FINDRISC and Cambridge risk scores.

**Conclusions:**

ID in apparently healthy middle-aged Mexican adults is currently at an alarming rate. The constructed models can be implemented to predict diabetes risk and represent the largest prospective effort for the study metabolic diseases in Latin-American population.

**Electronic supplementary material:**

The online version of this article (10.1186/s12902-019-0361-8) contains supplementary material, which is available to authorized users.

## Background

Type 2 diabetes (T2D)-related burden of disease in Mexico is among the biggest worldwide as there are currently over 9 million Mexicans living with diabetes [1]. T2D is among the top causes of morbidity, disability and mortality in Mexico [2]. Furthermore, Mexican-derived populations living in the US are among the ethnic groups with the highest risk of T2D and its complications [3]. Increased susceptibility for T2D is mainly explained by the interaction between genetic factors including Amerindian-specific risk alleles and chronic exposure to a positive caloric balance [4]. Thus, T2D prevention programs are an urgent need for the healthcare system in Mexico. Nevertheless, evidence of population-specific statistics is required for the design and implementation of such actions.

Prevalence data has been consistently collected in the National Health Surveys every 6 years since 1994 and the surveys have shown significant growth in T2D prevalence over the years [2, 5–8]. The 2006 National Health and Nutrition Survey (ENSANut 2006), reported a T2D prevalence of 14.4%, among which 7.1% were previously undiagnosed. The prevalence of T2D, based on the number of diagnosed cases, increased to 9.2% in 2012 and 9.4% in 2016 [8]. However, information about incident diabetes is scarce [9]. Diabetes prevention depends on the prompt identification and treatment of at-risk individuals [1–3], who are often detected through risk factor assessment [10]. Several risk factors have been previously reported in Mexicans; a previous report from the Mexico City study links increased body-mass index (BMI), abdominal obesity, impaired fasting glucose, advanced age and hypertension with increased risk of incident T2D [11–13]. The aim of this report is to inform the incidence rates of T2D and impaired fasting glucose (IFG) found in middle-aged apparently-healthy Mexican adults living in urban centers during a three-year follow-up period, in order to identify risk factors associated to T2D incidence and develop a predictive model for T2D in a high-risk population. Before the present study, longitudinal data to evaluate and predict T2D risk had been lacking, which posed limitations on risk factor prediction, estimation of the impact of prevention programs and generation of pharmaco-economic models. To the best of our knowledge, this is the first prospective study with large-enough sample to validate risk factors definitions for T2D prediction adjusted to our population.

## Research design and methods

### Study sample and research design

We performed a prospective observational cohort study including Mexican adults living in large urban settings of central Mexico including Mexico City, Cuernavaca, Leon, Toluca and Aguascalientes to evaluate incidence of T2D, arterial hypertension and cardiovascular disease, We aimed to identify risk factors associated to ID in order to develop a predictive model for ID in our population. The study sample was composed by apparently-healthy adults ≥20 years, with BMI ≥20 kg/m^2^, who resided for > 6 months in the evaluated city, and without plans to move to other city in the short term, whose grandparents and parents were born in Mexico. We excluded individuals with previously diagnosed diabetes, cardiovascular disease, cerebral vascular disease, incapacitated to lift themselves out of their home, pregnancy, alcoholism (≥10 servings of alcohol per week), acute stress event or any condition that could potentially endanger her life in the three following years. Participants were identified and evaluated at their workplaces (offices of the federal government or private companies) (*n* = 3246), homes (*n* = 189) or during a visit of a relative to a medical unit (*n* = 2709). The home-based component of the study sample was part of the “Mexican Study of Nutritional and Psychosocial Markers of Frailty”, a population-based cohort study designed to assess the nutritional and psychosocial determinants of frailty and its consequences on health of Mexican older adults living in Coyoacán in Mexico City [14].

All assessments were performed at morning, after a 9-12 h fasting period. The evaluation consisted in a clinical examination using standardized questionnaires, anthropometric measurements and a blood draw. Demographic information and a medical history, including personal and family history of the most common chronic diseases, were obtained. The evaluation included a 24-h diet recall, 7-day food frequency questionnaire, the three-factor eating questionnaire [15], the short version of the International physical activity questionnaire (IPAQ) [16] and for adults ≥50 years, an assessment of their functionality and depression. Participants were informed about their results and were advised to visit a primary care physician to seek for treatment if required. They were contacted after a three-year period (±6 months) and invited to repeat the evaluation using the same tools and methods. Multiple approaches were applied to cases that were not reachable at the place in which they were originally invited to participate, including phone calls, e-mail messages, telegrams, invitations through friends or relatives, and visits to the workplace. The response rate was 80.7% (*n* = 6166). The study was approved by the Ethics Committee of the Instituto Nacional de Ciencias Médicas y Nutrición and all participants signed an informed consent form.

### Laboratory measurements

All serum samples were kept frozen until processed in a central laboratory certified by the External Comparative Evaluation of Laboratories Program of the College of American Pathologists (Departamento de Endocrinología y Metabolismo, Instituto Nacional de Ciencias Médicas y Nutrición, México City). Clinical chemistry parameters and the lipid profile were measured using commercially available reagents (Synchron CX5 delta, Beckman Coulter). Immunonephelometric methods were applied for the measurement of apolipoprotein B (IMMAGE, Beckman Coulter) and C reactive protein (BN ProSpec, Siemens). Insulin concentrations were measured using an ELISA method (AxSYM, Abbott).

### Outcomes and variable definitions

Incident diabetes (ID) was defined if a previously healthy subject (fasting plasma glucose (FPG) < 126 mg/dL) at baseline had a medical diagnosis of T2D or started treatment with a glucose-lowering drug after follow-up and/or had a fasting glycemia ≥126 mg/dL in the second visit. Incident impaired fasting glucose (IFG) was defined by FPG in the range 100-125 mg/dL in the final visit for individuals that had the same variable < 100 mg/dL at baseline. Early-onset T2D was defined as T2D diagnosed < 40 years, as previously described [17]. Arterial hypertension was diagnosed according to the AHA guidelines [18]. Hypercholesterolemia was defined by the presence of a total cholesterol concentration > 200 mg/dL or being under statin therapy. Metabolic syndrome and its components were defined according to IDF and ATP-III recommendations [19].

### Statistical analyses

To evaluate inter-group differences, we used Student’s t and Mann-Whitney U tests, where appropriate. Frequency distribution of categorical variables were reported as frequencies and percentages and compared using chi-squared tests. For follow-up evaluations we used Student’s paired t and Wilcoxon’s rank-sign tests, where appropriate. Logarithmic transformations were applied to approximate normality in variables showing a non-parametric distribution. Missing values were imputed using Multiple Imputation by Chained Equations (MICE) and variables with > 5% of missing values were not included in the analyses. Data are presented as mean ± SD or as median and interquartile range.

Person-years for diabetes were calculated from baseline examination until the event or death occurred or until the last follow-up, whichever came first. Incidence of diabetes with 95%CI was calculated per 1000 person-years and risk factors were evaluated using unadjusted Cox proportional hazard regression models. To develop a risk score to predict ID in Mexican population, we fitted Cox proportional hazard regression models stratified by sex in two models: a first model comprising only demographic and anthropometric data and a model which also included biochemical measurements. β-coefficients from Cox regression models were used to develop a point-score for ID prediction, which was later validated using k-fold and bootstrap cross-validation to correct for over-optimism. Predictive performance of these models was evaluated using Harrerl’s *c-statistic* and Sommer’s D_xy_: the performance of our score was compared with FINDRISC and the Cambridge risk using non-parametric ROC tests. A two-tailed *p*-value< 0.05 was considered statistically significant. Statistical analyses were performed using the Statistical Package for Social Sciences software (SPSS, version 21.0), R software (Version 3.4.4) and GraphPad Prism version 6.0.

## Results

### Study population

**Fig. 1:**
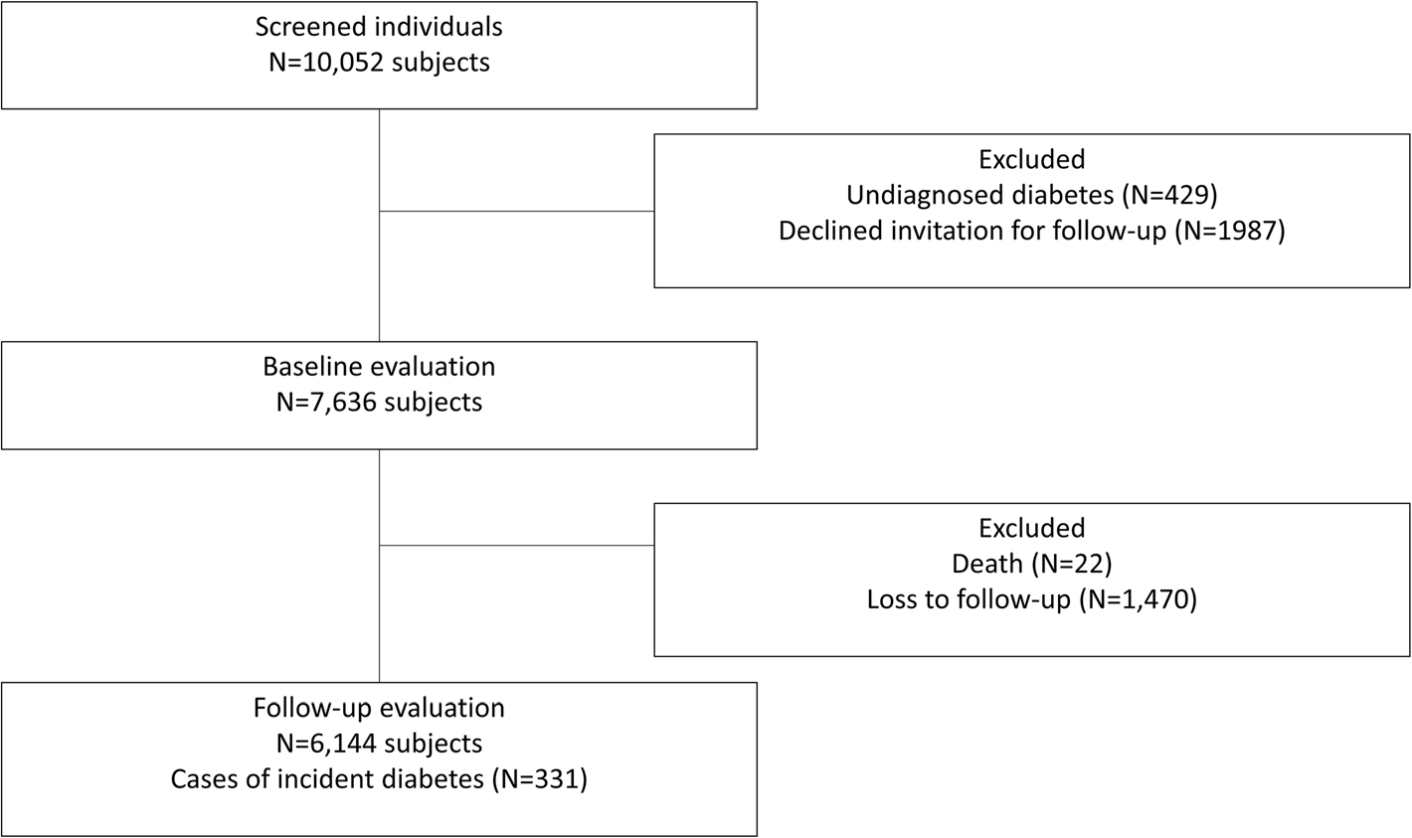
Flowchart of study participants at baseline and follow-up, outlining reasons for exclusion

Clinical data and blood samples were obtained from 10,052 individuals at baseline from 2007 to 2011. Among them, 2416 individuals had either undiagnosed T2D (*n* = 429) or declined permission to be included in the follow-up (*n* = 1987). Consequently, our study sample considered for the primary end-point of this report 7636 participants. The follow-up visit was performed 29.5 ± 9.7 months later (2010–2013); 6166 patients were reached for the second evaluation. Twenty-two deaths were recorded among participants. Therefore, 6144 subjects completed the second evaluation, comprising 15,501 person-years of follow-up (Fig. 1). Mexico City had the highest participation rate (*n* = 2493, 40.6%), followed by Aguascalientes (*n* = 1589, 25.9%), León (*n* = 997, 16.2%), Toluca (*n* = 864, 14.0%) and Cuernavaca (*n* = 201, 3.3%). The population is composed by middle-aged adults (42.6 ± 11.0 years), predominantly women (*n* = 4092, 66.6%), who had 12.1 ± 6.7 years of education. No significant differences were found in any of the socio-demographic or clinical parameters evaluated between study participants who completed or missed the follow-up visit. Our data confirmed a high prevalence of several metabolic abnormalities found in Mexicans. Abdominal obesity was found in 78.1%. The prevalence IDF-defined metabolic syndrome was 43.9%. Impaired fasting glucose was observed in 682 subjects (11.1%). The most common lipid abnormality was hypoalphalipoproteinemia, defined as HDL cholesterol< 40 mg/dL (59.8%).

### Diabetes incidence across subgroups

ID occurred in 331 cases (5.3%). In general, ID cases were older, had higher blood pressure, higher FPG, insulin, lipids, apolipoprotein B and C-reactive protein both at baseline and follow-up (Table 1). The incidence rate (IR) in the whole population was 21.9 cases per 1000 persons/year (95%CI 21.37–22.47) with a higher rate observed in men (IR 22.4, 95%CI 21.5–23.4) compared to women (IR 21.6 95%CI 21.0–22.3). At baseline, IFG was present in 682 cases at baseline (11.1%). Of them, 288 (42.2%) remained in the IFG category, 150 (22%) progressed to diabetes and 244 (35.8%) had an FPG < 100 mg/dL at the end of the follow-up. ID ranged from 13.6 cases per 1000 persons/year (95%CI 13.2–13.9) with glucose < 100 mg/dL to 162.74 cases per 1000 persons/year (95%CI 137.2–193.0) in the population with fasting blood glucose between 110 and 125 mg/dL. Among individuals with IFG at baseline (100–125.9 mg/dL), the incidence was 84.8 cases per 1000 persons/year (95%CI 78.7–91.4), a rate seven-fold higher compared to the rest of the population. ID was also proportional to BMI and we observed a four- fold difference in ID rates between lean persons and subjects with BMI > 35 kg/m^2^. Incidence rates were also higher in older subjects (≥55 years, 38.4 per 1000 persons/year, 95%CI 35.8–41.2) or with triglycerides > 150 mg/dL (29.0 per 1000 persons/year, 95%CI 28.0–29.9). Incidence rates observed in subsets of cases defined by age, gender, FPG and BMI are shown in Table 2.

**Table 1:** Baseline and follow-up biochemical and anthropometric characteristics comparing individuals who did and did not develop incidence diabetes after follow-up

Parameter	No diabetes (*N* = 5813)		*p*-value	Incident diabetes (*n* = 331)		*p*-value
	Baseline	Follow-up		Baseline	Follow-up	
Metabolic syndrome IDF (%)	2455 (42.2%)	2673 (46.0%)	<0.001	244 (73.7%)	271 (81.9%)	<0.001
Metabolic syndrome ATP-III (%)	1821 (31.3%)	2040 (35.1%)	<0.001	217 (65.6%)	276 (83.1%)	<0.001
Fasting glucose (mg/dL)	85.35 ± 10.45	85.53 ± 11.89	0.227	97.47 ± 13.42	112.69 ± 38.41	<0.001
BMI (kg/m^2^)	28.65 ± 4.58	28.74 ± 4.66	0.001	31.01 ± 5.19	30.79 ± 5.18	0.061
Waist circumference (cm)	92.72 ± 11.44	93.57 ± 11.47	<0.001	98.14 ± 12.40	98.92 ± 12.70	0.064
Waist-hip ratio	0.88 (0.83–0.94)	0.89 (0.84–0.94)	<0.001	0.91 (0.86–0.98)	0.91 (0.86–0.96)	0.482
Waist-height ratio	0.57 (0.53–0.62)	0.58 (0.54–0.62)	<0.001	0.60 (0.57–0.65)	0.61 (0.57–0.67)	0.086
Fasting triglycerides (mg/dL	187.90 ± 141.26	174.20 ± 112.42	<0.001	226.93 ± 154.87	207.59 ± 121.16	0.020
Fasting insulin (μI/mL)	11.75 ± 7.64	12.11 ± 10.14	0.007	15.82 ± 10.30	17.26 ± 15.37	0.083
Total cholesterol (mg/dL)	205.76 ± 40.94	197.78 ± 40.07	<0.001	211.17 ± 40.69	202.69 ± 39.33	<0.001
HDL-c (mg/dL)	44.81 ± 11.69	41.71 ± 12.11	<0.001	42.41 ± 10.97	40.20 ± 10.88	<0.001
LDL-c (mg/dL)	125.48 ± 32.09	122.64 ± 31.34	<0.001	128.91 ± 32.61	126.18 ± 30.42	0.166
Non-HDL-c (mg/dL)	160.94 ± 39.31	156.06 ± 37.87	<0.001	168.76 ± 37.86	162.48 ± 36.50	<0.001
Apolipoprotein B (mg/dL)	108.27 ± 26.95	103.05 ± 26.11	<0.001	114.92 ± 26.00	108.42 ± 27.60	<0.001
C-reactive protein	1.88 (0.97–3.91)	1.82 (0.86–3.81)	0.057	2.97 (1.45–5.32)	3.04 (1.46–6.10)	0.689

*P*-values for paired comparisons in each group

**Table 2:** Diabetes incidence rates (cases/1000 persons per year) in the study sample stratified by gender, age and impaired fasting glucose

Sex	Category	< 35	35–44.9	45–54.9	≥55
Males	BMI < 25 kg/m^2^	12.66	9.61	8.85	13.60
	BMI 25–29.9 kg/m^2^	6.45	14.06	10.60	43.26
	BMI ≥30 kg/m^2^	12.22	26.86	43.66	44.44
	BMI < 25 kg/m^2^ + FPG < 100 mg/dl	12.86	6.97	4.93	–
	BMI < 25 kg/m^2^ + FPG ≥100 mg/dl	–	40.00	43.48	74.07
	BMI 25–29.9 kg/m^2^ + FPG < 100 mg/dl	4.09	7.80	7.21	23.03
	BMI 25–29.9 kg/m^2^ + FPG ≥100 mg/dl	47.62	71.43	36.70	112.36
	BMI ≥30 kg/m^2^ + FPG < 100 mg/dl	13.23	8.93	29.89	29.41
	BMI ≥30 kg/m^2^ + FPG ≥100 mg/dl	–	118.18	92.59	90.91
Females	BMI < 25 kg/m^2^	6.68	4.30	13.77	18.25
	BMI 25–29.9 kg/m^2^	15.68	10.24	16.74	33.97
	BMI ≥30 kg/m^2^	18.61	21.47	36.27	28.71
	BMI < 25 kg/m^2^ + FPG < 100 mg/dl	5.42	4.46	10.83	4.27
	BMI < 25 kg/m^2^ + FPG ≥100 mg/dl	100.00	–	74.07	100.00
	BMI 25–29.9 kg/m^2^ + FPG < 100 mg/dl	13.66	8.32	13.87	11.56
	BMI 25–29.9 kg/m^2^ + FPG ≥100 mg/dl	60.00	39.06	40.54	107.59
	BMI ≥30 kg/m^2^ + FPG < 100 mg/dl	11.09	11.44	23.15	20.64
	BMI ≥30 kg/m^2^ + FPG ≥100 mg/dl	82.35	81.90	86.06	47.12

Abbreviations: *FPG* Fasting plasma glucose, *BMI* Body-mass index

The rates of incident IFG were greater compared to ID. Incident IFG occurred in 450 cases (8.1% of the normoglycemic population at baseline). The incidence rate in the whole population was 25.59 cases per 1000 persons/year. Higher rates were observed in men (27.4 vs 24.7 per 1000/year) and in subjects older than age 55, BMI ≥ 35 kg/m^2^ or triglycerides > 150 mg/dL. The highest IFG incidence rates were observed in subjects with FPG ≥90 mg/dL. Young obese subjects had similar IFG incident rates than those observed in lean individuals older than age 55.

### Anthropometric and sociodemographic risk factors for ID

We observed a higher risk of ID in first-degree relatives of T2D cases. Furthermore, we observed higher ID risk for individuals ages 45–60 (HR 1.89 95%CI 1.25–2.84) and > 60 years (HR 2.20 95%CI 1.33–3.64) compared to the reference group (Table 3). In addition, we identified significantly higher risk of ID with abdominal obesity by IDF criteria, which was higher in men (HR 2.45 95%CI 1.37–4.37) compared to women (HR 1.98 95%CI 1.30–3.03). Abdominal obesity by ATP-III criteria was also associated, though the risk was lower. Overweight and obese BMI categories were also associated with higher ID risk in comparison to normal BMI group. When evaluating other anthropometric measures, we observed an increased risk for waist-hip (WH) ratios > 0.85 in females and > 0.90 in males and the waist-height ratio (WHtr) > 0.5; using ROC curves, we observed the highest AUC for the WHtr, which also had the highest risk amongst all other anthropometric indexes (Additional file 1: Table S1). When assessed the role of gender-based differences in ID risk, we observed no significant differences between men and women (*p* = 0.418), but we did observe a significant interaction of sex with increasing age. Compared to women < 40 years, men aged 55–70 (HR 1.94 95%CI 1.20–3.16) and men > 70 years (HR 3.20 95%CI 1.31–7.83) had higher rates if ID adjusted for WC, family history of T2D, physical activity and smoking. Among women, the ID risk was higher in post-menopausal women (HR 1.39 95%CI 1.02–2.24) adjusted for hypertension, family history of T2D, WC, physical activity and smoking.

**Table 3:** Assessment of anthropometric, demographic and biochemical risk factors for incident diabetes obtained through unadjusted Cox-proportional hazard regression analyses in Mexican population according to predefined cut-off values

Parameter	β	HR	95%CI	*P*-value
Age ≥ 40 years	0.537	1.711	1.345–2.175	<0.001
Family history of T2D	0.287	1.332	1.069–1.660	0.011
Waist circumference (IDF)	0.918	2.505	1.677–3.744	<0.001
Waist circumference (ATP-III)	0.660	1.934	1.539–2.430	<0.001
Overweight 25–29.99 kg/m^2^	0.453	1.572	1.068–2.316	0.022
Obesity (≥30 kg/m2)	0.902	2.464	1.685–3.605	<0.001
High Waist-hip ratio^a^	0.542	1.720	1.346–2.198	<0.001
Waist-height index > 0.5	0.983	2.673	1.499–4.767	<0.001
Blood pressure > 140/90 mmHg	0.672	1.958	1.566–2.447	<0.001
Fasting glucose 100–110 mg/dL	1.470	4.347	3.378–5.594	<0.001
Fasting glucose 111–125 mg/dL	2.353	10.512	7.792–14.182	<0.001
Fasting insulin ≥15uUI/L	0.634	1.886	1.501–2.368	<0.001
HOMA2-IR > 2.5	0.871	2.389	1.877–3.042	<0.001
Fasting triglycerides > 150 mg/dL	0.824	2.280	1.767–2.943	<0.001
Total colesterol > 200 mg/dL	0.391	1.478	1.174–1.861	0.001
Low HDL-C	−0.225	0.798	0.636–1.003	0.053
Non-HDL-C > 130 mg/dL	0.599	1.820	1.325–2.500	<0.001
Apolipoprotein B (>90th percentile)	0.415	1.515	1.205–1.903	<0.001
CPR ≥2.3	0.470	1.600	1.278–2.003	<0.001

^a^Waist hip ratio > 0.85 females, > 0.9 males

### Metabolic risk factors for ID

Among biochemical variables, the strongest predictor of ID was FPG. Subjects with FPG 100-110 mg/dL had four-fold higher risk of ID compared to subjects with FPG < 100 mg/dL, with the highest risk attributable to FPG 111–125 mg/dL. Overall, subjects with IFG had five-fold higher risk of ID compared to normoglycaemic subjects. We also observed a significant interaction between age and fasting glycaemia to predict ID cases (*p* < 0.001). Subjects with IFG aged 30–45 years had higher risk of ID compared to individuals < 30 years (HR 5.40 95%CI 4.0–7.29), an observation that was also confirmed in the 46–60 (HR 5.71 95%CI 4.31–7.56) and > 60 year-groups (HR 7.09 95%CI 4.46–11.26). The predictive capacity of each biochemical measure according to pre-defined cut-offs showed the highest ID risk for HOMA2-IR > 2.5 and triglycerides > 150 mg/dL (Table 3).

### Metabolic syndrome and ID

**Fig. 2:**
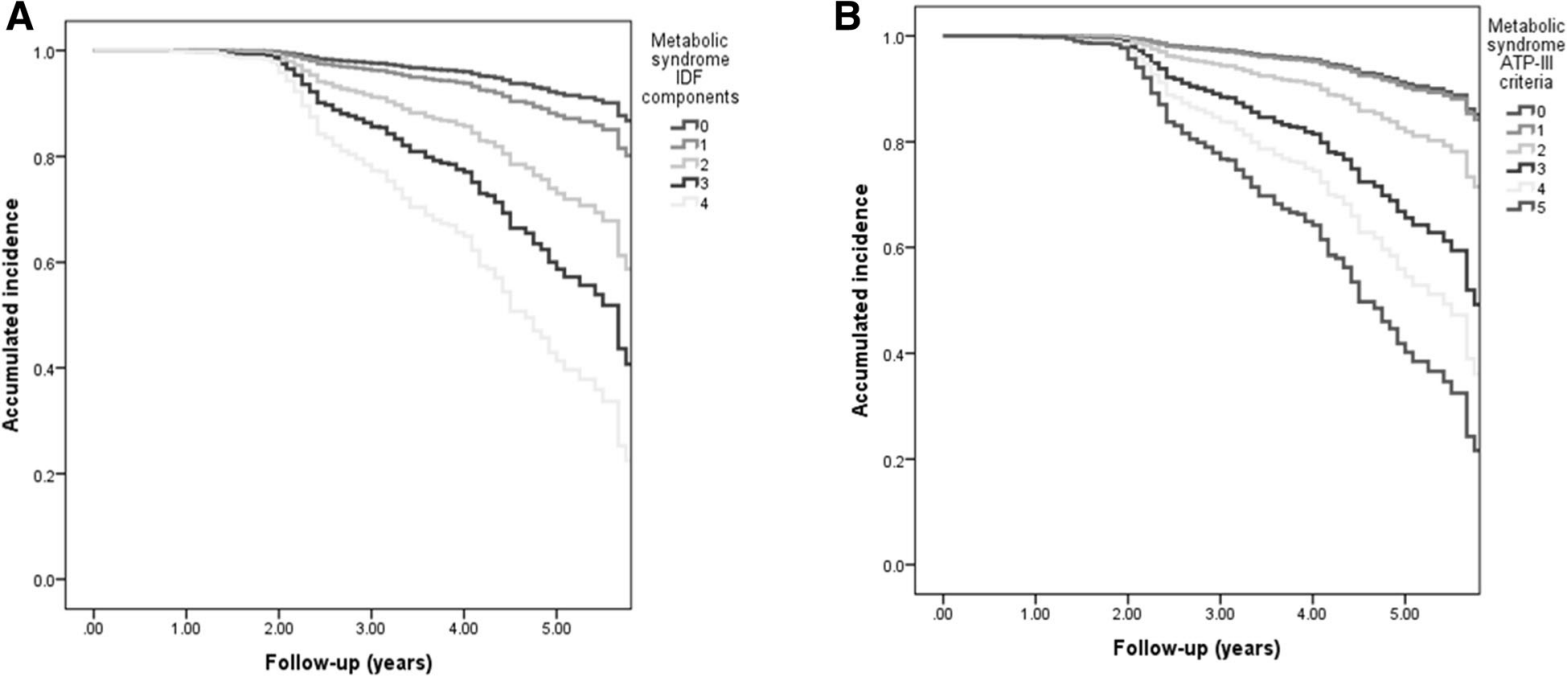
Kaplan-Meier survival analyses for incident diabetes according to the number of components based on IDF (**a**) and ATP-III (**b**) criteria adjusted for age and sex. In both IDF and ATP-III criteria, we observed a significantly higher accumulated incidence for each additional MS component > 1 (*p* < 0.001) compared to none of the risk factors

We observed a three-fold higher ID risk in subjects who had metabolic syndrome by IDF criteria (MS-IDF) at baseline (HR 3.42, 95%CI 2.68–4.37) compared to those who did not. ID risk was higher using the ATP-III criteria MS definition (MS-ATP-III, HR 1.81 95%CI 1.72–2.13). In relation to MS-IDF criteria, we observed significantly higher risk with ≥2 components. We observed a higher risk with 2 components (HR 3.84 95%CI 2.21–6.68), 3 components (HR 6.76 95%CI 3.86–11.85) and the highest with 4 components (HR 11.59 95%CI 6.29–21.37). Using MS-ATP-III the risk increased with 2 components (HR 2.15 95%CI 1.17–3.97), 3 components (HR 4.52 95%CI 2.49–8.21), 4 components (HR 6.84 95%CI 3.72–12.59) and 5 components (HR 10.43 95%CI 5.32–20.45), which was lower compared to MS-IDF (Fig. 2).

### Risk factors for early-onset incident diabetes

We observed 93 cases of early onset ID over 6298-person years, yielding an incidence rate of 14.77 cases per 1000 person-years (95%CI 14.21–15.35), which was lower to that observed in individuals with ID onset > 40 years (IR 27.02 95%CI 26.14–27.92). At baseline, subjects with early-onset ID had higher HOMA-IR, fasting insulin, triglycerides compared to subjects with ID ≥40 years. Furthermore, subjects with early-onset ID had lower FPG, BMI, waist circumference, systolic and diastolic blood pressure, total cholesterol, HDL-C and apoB levels, adjusted for age and sex. Using multivariate Cox regression, we observed that HOMA-IR > 2.5 (HR 1.82 95%CI 1.13–2.93) and FPG > 100 mg/dL (HR 2.26 95%CI 1.63–3.14) were risk factors for early onset ID, whilst physical activity was a protective factor (HR 0.55 95%CI 0.36–0.83), adjusted for age, sex, first-degree family history of diabetes, WHtr > 0.5, smoking and hypertension. Finally, we observed a statistically significant interaction between HOMA-IR > 2.5 and first-degree family history of T2D (HR 1.79 95%CI 1.05–3.04) only in individuals with early onset ID. For ID in individuals ≥40 years, risk factors included hypertension (HR 1.47 95%CI 1.11–1.94), WHtr > 0.5 (HR 1.82 95%CI 1.27–2.61) and FPG > 100 mg/dL (HR 3.17 95%CI 2.66–3.79). Physical activity and insulin resistance estimated using HOMA-IR were not associated with ID in individuals > 40 years.

### Development of a predictive model for diabetes incidence

We developed two main models for prediction of ID in Mexican population, an office-based model, which does not rely on fasting laboratory measurements, and a clinical biochemical method. For the office-based model, we identified as potential predictors age > 40 years, first-degree family history of T2D, WHtr > 0.5, arterial hypertension and BMI ≥ 30 kg/m^2^ (Table 4); the model was validated using k-fold cross-validation (k = 10) and bootstrap validation (D_xy_ = 0.287, *c-statistic* = 0.656). We constructed a point-based model using β-coefficients assigning a score = 1.0 to β-coefficients < 0.35, 2 to β-coefficients 0.35–0.7 and 3 to coefficients > 0.7. Using Cox regression, we evaluated the predictive capacity of threshold scores for ID. Using as reference level scores 1–3, scores between 4 and 6 had nearly two-fold higher risk for ID (HR 1.87 95%CI 1.18–2.98), followed by scores 7–8 (HR 3.36 95%CI 2.11–5.37) and the highest risk for scores 9–10 (HR 5.43 95%CI 3.31–8.91). Accumulated incidence was different between score categories (log-rank *p* < 0.001).

**Table 4:** Office-based and biochemical model for prediction of incident diabetes from Cox-proportional hazard regression models

Model parameters	Variable	β-coefficient	Wald	HR	95%CI	*p*-value	Points
Office-based model X^2^ = 76.64 *p* < 0.0001 D_xy_ = 0.2915 c-statistic = 0.656	Age > 40 years	0.466	14.079	1.593	1.249–2.031	<0.001	2
	FDFH of T2D	0.242	4.560	1.273	1.020–1.590	0.033	1
	WHr > 0.5	0.725	5.809	2.065	1.145–3.725	0.016	3
	Arterial hypertension	0.503	18.471	1.654	1.315–2.080	<0.001	2
	BMI ≥30 kg/m2	0.388	11.500	1.474	1.178.1.845	0.001	2
Biochemical model X^2^ = 446.815 p < 0.0001 D_xy_ = 0.4723 c-statistic = 0.752	Physical activity	−0.217	3.374	0.805	0.638–1.015	0.066	−1
	Age > 40 years	0.328	6.830	1.388	1.085–1.776	0.009	2
	TG > 150 mg/dL	0.517	14.932	1.677	1.287–2.185	<0.001	3
	Glucose 100–110 mg/dL	1.271	92.355	3.565	2.751–4.621	<0.001	4
	Glucose 111–125 mg/dL	2.097	176.167	8.138	5.971–11.091	<0.001	7
	Arterial hypertension	0.306	6.711	1.358	1.077–1.712	0.010	2
	Abdominal obesity (IDF)	0.422	5.576	1.525	1.074–2.165	0.018	2

Discrimination indexes from both regression models were obtained from k-fold cross-validation (k = 10) and were corrected for over-optimism

**Fig. 3:**
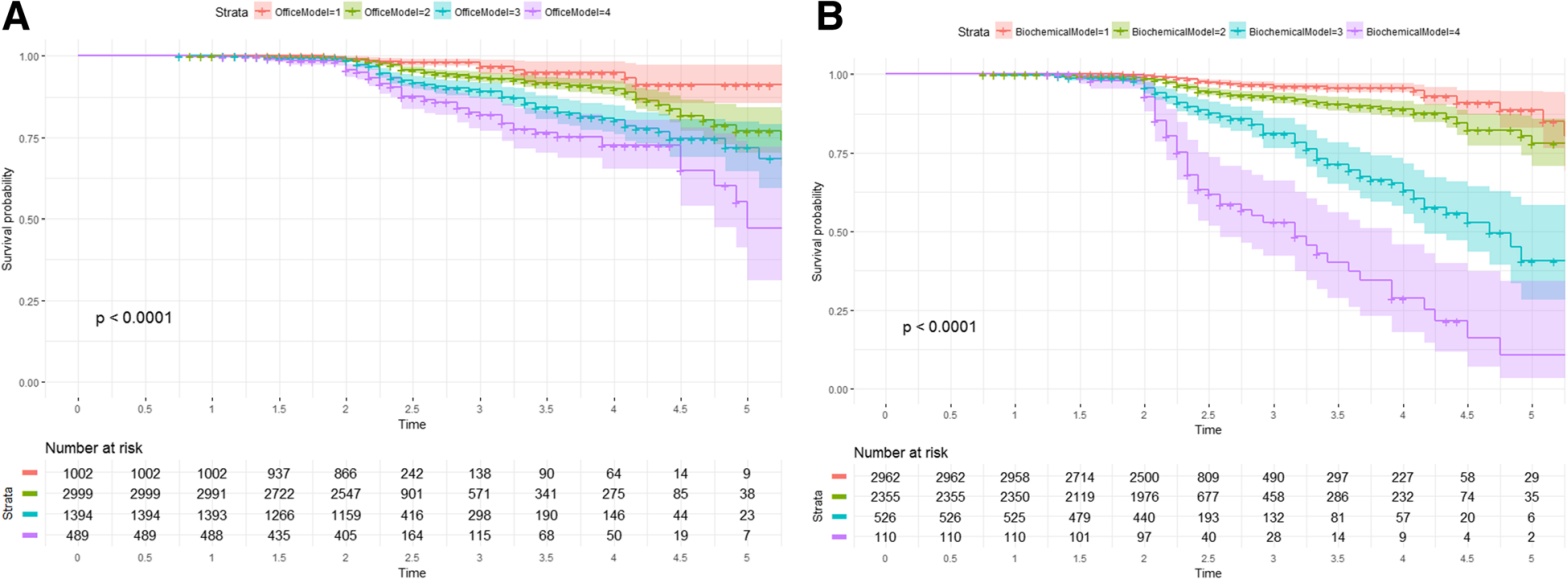
Kaplan-Meier survival curves for incident diabetes according to stratified scores by quartiles obtained from our office-based and biochemical models for incident diabetes in the MS cohort (log-rank test *p* < 0.001)

For the biochemical model, we identified as potential predictors age > 40 years, fasting triglycerides > 150 mg/dL, FPG 100–110 mg/dL, FPG 111–125 md/dL, arterial hypertension and abdominal obesity as diagnosed by IDF criteria, which was also validated and corrected for over-optimism (D_xy_ = 0.487, *c-statistic* = 0.741). Next, we constructed a similar model, assigning scores using a similar methodology from the office-based model. We analyzed strata using Cox regression and using as a reference scores > − 1 but ≤4 we observed increased risk in patients with scores 5–8 (HR 2.28 95%CI 1.68–3.10), followed by scores 9–12 (HR 6.99 95%CI 5.04–3.69) and the highest risk for scores 13–16 (HR 18.69 95%CI 12.83–27.22). Evaluation between score categories showed different accumulated incidence (log-rank p < 0.001, Fig. 3). Overall, the biochemical model had a higher predictive accuracy (AUC = 0.752 95%CI 0.724–0.781), compared to FINDRISC (AUC = 0.634 95%CI 0.604–0.664) and the Cambridge risk score (AUC 0.654 95%CI 0.623–0.686) in our population.

## Discussion

Our work is the first to estimate T2D incidence in central Mexico and the first in Latin America with sample large enough to develop predictive models in a high-risk, genetically-predisposed population. The only previous report about ID in adult Mexicans reported that 7% of 1244 adults who resided in a Mexico City neighborhood had hyperglycemia during a six-year observational period [9]. Even though direct comparisons between studies are not feasible, the incidence reported in our population is higher considering follow-up time, which reported 5.38% in a median of 2.4 years. FPG was the variable with the highest predictive value, followed by the WHtr, obesity diagnosed by BMI, hypertriglyceridemia > 150 mg/dL and HOMA2-IR values > 2.5. Despite the fact that FPG has been questioned as a detection method for type 2 diabetes, in our population it was a major prognostic factor for T2D.

The increase in diabetes-related mortality and the poor metabolic control in diagnosed individuals in Mexico represents a major concern [20]. Identifying risk factors for incident diabetes is of paramount importance for early detection of at-risk individuals, especially considering that T2D often has early-onset in our population, which leads to a higher incidence of adverse metabolic and cardiovascular outcomes [2, 8].

Several prognostic models and scores for type 2 diabetes risk have been developed based on identified risk factors including age, sex, obesity, diet, exercise, ethnicity, family history of diabetes amongst others. Our findings are similar to the FINDRISC study in Finland [21], which also included BMI, age and physical activity. However, the application of the FINDRISC score in our population does not have a high predictive accuracy. Our biochemical model was decidedly superior. The Australian AUSDRISK study [22] and UK-based Cambridge Risk Score, [23] also include age, sex, family history of diabetes, BMI and physical activity also underperformed in comparison to the biochemical model but were superior to the office-based model. The model reported here outscores other models (i.e. those derived from the ARIC [24] and the Framingham Offspring Study [25]), which include family history of diabetes and age and strongly differ from our proposed models.

Diabetes incidence in our study was among the highest reported in the literature for different ethnic groups, particularly considering the relatively short follow-up period. This high diabetes incidence could be attributable to the elevated prevalence of overweight and obesity across different age ranges in Mexican population as well as the high rate of inactivity combined with a high carbohydrate and fat intake. As reported by Stolerman et al., incorporation of genetic risk scores does not improve the prognostic performance of predictive models including clinical variables in a multiethnic cohort, which suggests that environmental risk factors could have a much greater impact in diabetes development in interaction with genetic risk factors [26]. Currently, there are several efforts to integrate -omics- technologies in risk prediction, which should be helpful to increase predictive performance of risk models with potential biomarkers of risk including genetic variants, RNA transcripts, peptides, lipids, small metabolites, cell markers and metabolic-driven products [27].

Our study had some strengths and limitations. First, we evaluated a large prospective effort to estimate diabetes incidence in a high-risk, not previously evaluated population, which allowed for identification of metabolic risk factors that predict ID. The loss to follow-up was relatively minor (19.6%), with no significant differences comparing individuals who did and did not complete follow-up, which allowed for an adequate estimate of diabetes incidence with enough statistical power to develop predictive models and validate metabolic measures [28]. Furthermore, we validated both our models using k-fold cross-validation and bootstrap to correct for over-optimism, which ensures validity of our observations. We also evaluated our proposed score against competing models constructed with similar variables and observed a superior predictive performance. The main limitations to be recognized is the lack of an external validation for calibration of the risk scores, which calls for further evaluations to assess the validity to implement our score in other Latin American populations. In addition, the inclusion criteria for this study could generate bias towards subjects with the highest risk, which calls for additional evaluations in low-risk populations with similar genetic profiles. Finally, given that T2D diagnosis was mainly based on previous diagnosis and a single abnormal FPG measurement, the true number of ID cases could have been underestimated if patients with undiagnosed T2D had FPG below the diagnostic threshold.

## Conclusion

Type 2 diabetes incidence in apparently healthy middle-aged Mexican adults residing in urban centers in Mexico is currently at an alarming rate. FPG is the strongest predictor of incident diabetes, particularly in overweight and obese individuals. We constructed two models that can easily be implemented to predict diabetes risk in Mexican population, including age, BMI, WHtr, IFG, arterial hypertension, fasting hypertriglyceridemia and family history of diabetes, which represents an advantage given their availability in primary-care facilities and allows for large-scale implementations. Further studies are required for validation of these models in similar at-risk populations. Our study represents the largest prospective study regarding metabolic diseases in Latin-American population and the only current predictive model for diabetes in Mexican population.

## Additional file

Additional file 1: **Table S1.** Assessment of anthropometric, demographic and biochemical risk factors for incident diabetes obtained through Cox-proportional hazard regression analyses in Mexican population, along with their predictive performance using area under the receiving operating characteristic curves. (DOCX 16 kb)

## Abbreviations

95%CI: 95% confidence interval
ATP-III: Adult treatment panel III
AUC: Area under the curve
BMI: Body-mass index
FINDRISC: Finnish Diabetes Risk Score
FPG: Fasting plasma glucose
HDL-C: High-density lipoprotein cholesterol
HOMA2-IR: Homeostatic model assessment for insulin resistance 2
HR: Hazard ratio
ID: Incident type 2 diabetes mellitus
IDF: International Diabetes Federation criteria
IFG: Impaired fasting glucose
IR: Incidence rates
MS: Metabolic syndrome
T2D: Type 2 Diabetes Mellitus
WHr: Weight to hip ratio
WHtr: Weight to height ratio
