# Supplementary material for: Development and validation of a predictive model for incident type 2 diabetes in middle-aged Mexican adults: the metabolic syndrome cohort
Source: BMC Endocr Disord. 2019 Apr 28;19:41. doi: 10.1186/s12902-019-0361-8 (PMC6486953; doi:10.1186/s12902-019-0361-8)
Supplement: Supplementary file 1 — Table S1. Assessment of anthropometric, demographic and biochemical risk factors for incident diabetes obtained through Cox-proportional hazard regression analyses in Mexican population, along with their predictive performance using area under the receiving operating characteristic curves. (DOCX 16 kb) [file 12902_2019_361_MOESM1_ESM.docx]

**Additional file 1: Table S1.** Assessment of anthropometric, demographic and biochemical risk factors for incident diabetes obtained through Cox-proportional hazard regression analyses in Mexican population, along with their predictive performance using area under the receiving operating characteristic curves.

| **Parameter** | **β** | **HR** | **95%CI** | **p** | **AUC (95%CI)** |
| --- | --- | --- | --- | --- | --- |
| Age | 0.024 | 1.025 | 1.015-1.034 | <0.001 | 0.599 (0.568-0.631) |
| Family history of T2D | 0.287 | 1.332 | 1.069-1.660 | 0.011 | 0.550 (0.519-0.582) |
| Waist circumference | 0.024 | 1.024 | 1.017-1.032 | <0.001 | 0.623 (0.593-0.654) |
| BMI | 0.059 | 1.060 | 1.042-1.079 | <0.001 | 0.639 (0.609-0.670) |
| Waist-hip ratio | 2.349 | 10.480 | 4.350-25.251 | <0.001 | 0.599 (0.568-0.630) |
| Waist-height index | 2.898 | 18.137 | 8.148-40.371 | <0.001 | 0.642 (0.613-0.672) |
| SBP | 0.018 | 1.019 | 1.012-1.025 | <0.001 | 0.587 (0.553-0.621) |
| DBP | 0.030 | 1.031 | 1.019-1.042 | <0.001 | 0.562 (0.529-0.596) |
| Glucose | 0.076 | 1.079 | 1.070-1.089 | <0.001 | 0.762 (0.731-0.792) |
| Insulin | 0.029 | 1.030 | 1.023-1.037 | <0.001 | 0.635 (0.603-0.667) |
| HOMA2-IR | 0.291 | 1.338 | 1.256-1.426 | <0.001 | 0.648 (0.616-0.679) |
| Triglycerides | 0.001 | 1.001 | 1.001-1.002 | <0.001 | 0.611 (0.582-0.641) |
| Total colesterol | 0.003 | 1.003 | 1.001-1.005 | 0.014 | 0.551 (0.518-0.583) |
| HDL-C | 0.017 | 0.983 | 0.973-0.993 | 0.001 | 0.566 (0.534-0.597) |
| Non-HDL-C | 0.004 | 1.004 | 1.002-1.007 | <0.001 | 0.567 (0.536-0.598) |
| Apolipoprotein B | 0.007 | 1.007 | 1.004-1.011 | <0.001 | 0.575 (0.544-0.606) |
| CPR | 0.088 | 1.092 | 1.053-1.133 | <0.001 | 0.602 (0.570-0.634) |
